# Single-Stage Externalized Locked Plating for Treatment of Unstable Meta-Diaphyseal Tibial Fractures

**DOI:** 10.3390/jcm12041600

**Published:** 2023-02-17

**Authors:** Biser Makelov, Dominic Mischler, Peter Varga, Theerachai Apivatthakakul, James W. A. Fletcher, Deyan Veselinov, Till Berk, Dimitur Raykov, Boyko Gueorguiev

**Affiliations:** 1 University Multiprofile Hospital for Active Treatment, Trakia University, 6003 Stara Zagora, Bulgaria; 2 AO Research Institute Davos, 7270 Davos, Switzerland; 3 Department of Orthopaedics, Faculty of Medicine, Chiang Mai University, Chiang Mai 50200, Thailand; 4 Department for Health, University of Bath, Bath BA2 7AY, UK; 5 Bulgarian Academy of Sciences, Institute of Metal Science ‘Acad. A. Balevski’, 1574 Sofia, Bulgaria; 6 University Hospital Zurich, 8091 Zurich, Switzerland; 7 Department of Orthopaedics and Traumatology, Faculty of Medicine, Medical University Varna, 9002 Varna, Bulgaria

**Keywords:** externalized locked plating, supercutaneous plating, unstable tibial fractures

## Abstract

(1) Background: Unstable meta-diaphyseal tibial fractures represent a heterogeneous group of injuries. Recently, good clinical results have been reported when applying a technique of externalized locked plating in appropriate cases, highlighting its advantage in terms of less additional tissue injury compared with conventional methods of fracture fixation. The aims of this prospective clinical cohort study were, firstly, to investigate the biomechanical and clinical feasibility and, secondly, to evaluate the clinical and functional outcomes of single-stage externalized locked plating for treatment of unstable, proximal (intra- and extra-articular) and distal (extra-articular), meta-diaphyseal tibial fractures. (2) Methods: Patients, who matched the inclusion criteria of sustaining a high-energy unstable meta-diaphyseal tibial fracture, were identified prospectively for single-stage externalized locked plating at a single trauma hospital in the period from April 2013 to December 2022. (3) Results: Eighteen patients were included in the study. Average follow-up was 21.4 ± 12.3 months, with 94% of the fractures healing without complications. The healing time was 21.1 ± 4.6 weeks, being significantly shorter for patients with proximal extra- versus intra-articular meta-diaphyseal tibial fractures, *p* = 0.04. Good and excellent functional outcomes in terms of HSS and AOFAS scores, and knee and ankle joints range of motion were observed among all patients, with no registered implant breakage, deep infection, and non-union. (4) Conclusions: Single-stage externalized locked plating of unstable meta-diaphyseal tibial fractures provides adequate stability of fixation with promising clinical results and represents an attractive alternative to the conventional methods of external fixation when inclusion criteria and rehabilitation protocol are strictly followed. Further experimental studies and randomized multicentric clinical trials with larger series of patients are necessary to pave the way of its use in clinical practice.

## 1. Introduction

Unstable, meta-diaphyseal tibial fractures represent a heterogeneous group of injuries often caused by high-energy trauma, such as during road traffic accidents or falls from height [1,2,3]. With a prevalence between 2% and 11%, commonly these injuries result in disability, with high socioeconomic costs [4,5,6]. Due to their high variability and compromised soft tissue envelope, standardized treatment algorithms are difficult to apply, posing a treatment challenge for orthopedic surgeons [6,7,8,9]. In polytrauma patients—especially those with considerable local soft tissue damage—the proper timing of adequate early definitive treatment is crucial. Standard surgical methods for definitive fixation include use of locking plates, intramedullary nails, and/or external fixation devices [10,11,12,13,14,15,16]. However, there are problems related to the: (1) selection of an appropriate surgical technique to avoid additional soft tissue injury at the fracture site, (2) choice between a staged or single-stage approach, (3) optimum timing for intervention, and (4) complexity and duration of the operative intervention. Initially, an operative technique using a locking plate as an external fixator for treatment of open tibial fractures—called supercutaneous locked plating or externalized locked plating—was described and used to address some of the concerns regarding operative approaches in the context of soft tissue injuries [17,18,19]. Recently, good clinical outcomes with positive therapeutic results have been reported when applying this technique in properly indicated cases, highlighting its advantage in terms of less additional tissue injury and sufficient axial and torsional stability when compared with conventional methods of fracture fixation [19,20,21,22,23,24,25,26,27,28,29,30,31,32]. Moreover, experimental and virtual biomechanical studies have investigated the stability and endurance of supercutaneously plated constructs with different fracture gap sizes and plate elevations versus monolateral conventional external fixations to conclude that (1) the stiffness and elasticity of the former depends on the implant elevation, and therefore, the externalized locked plating requires a low-profile design with implant placement as close as possible to the skin, and (2) with appropriate plate elevation, the interfragmentary strain in the fracture gap under immediate partial weight bearing (PWB) is within the range indicated for successful secondary fracture healing [21,33,34,35,36,37,38]. Previous work has shown how using a pre-contoured Less-Invasive Stabilization System Distal Femur (LISS-DF) plate as a definitive monolateral external splinting device for fixation of a multi-fragmentary, proximal, meta-diaphyseal tibial fracture in a polytrauma patient led to uneventful fracture healing, without the occurrence of deep infection, screw loosening, or breakage, and with implant removal without any complications in outpatient facilities [39]. Building on this, we felt investigation was required into the use of a single-stage externalized locking plate (SSELP) in the management of unstable tibial fractures.

The aims of this prospective cohort study were, firstly, to investigate the biomechanical and clinical feasibility of using SSELP and, secondly, to evaluate the clinical and functional outcomes following the application of SSELP for treatment of unstable, proximal (intra- and extra-articular) and distal (extra-articular), meta-diaphyseal tibial fractures.

## 2. Materials and Methods

The study was approved by the Institutional Review Board (Approval 11/27 June 2019). Signed informed consent was obtained from all participants.

### 2.1. Patients

**Figure 1:**
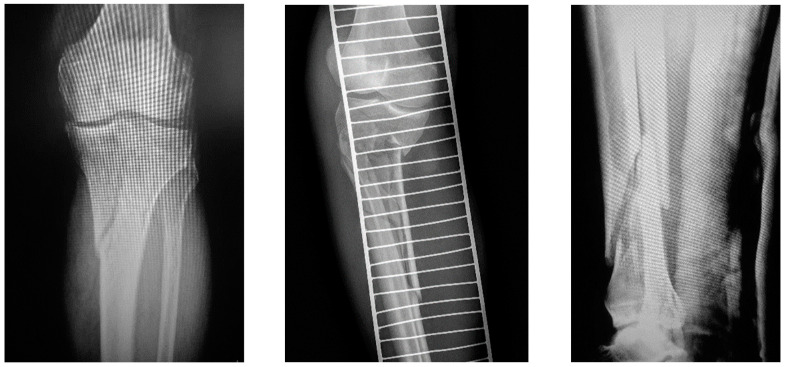
Preoperative radiographs of patients with an AO/OTA 41-C2.2 proximal tibial fracture with simple intra-articular involvement of both tibial condyles without intra-articular comminution and impaction (**left**), a 41-A3.1 multi-fragmentary proximal tibial extra-articular fracture (**middle**), or a 43-A2.3 distal tibial multi-fragmentary meta-diaphyseal fracture (**right**), all accompanied by significant soft tissue injury.

Patients with unstable, meta-diaphyseal tibial fractures, with or without intra-articular involvement, caused by high-energy trauma, and treated with SSELP with the use of LISS-DF in the period from April 2013 to December 2022 were considered for inclusion. Inclusion criteria were patient age above 18 years, as well as radiologically confirmed diagnosis of either multi-fragmentary proximal tibial extra-articular fractures, proximal tibial intra-articular fractures with simple intra-articular involvement of both tibial condyles without intra-articular comminution and impaction, distal tibial multi-fragmentary extra-articular meta-diaphyseal fractures with significant soft tissue injury or multiple trauma, or open tibial fractures with either suspected severe wound contamination or a ‘floating knee injury’ (Table 1, Figure 1). Exclusion criteria considered patients with either low-energy fractures with intact soft tissue coverage, fractures representing critical-size bone defects or aggregate defects greater than 20 mm, avulsion AO/OTA 42-A fractures, isolated intra-articular AO/OTA 41-B tibial plateau fractures, or complex multi-fragmentary intra-articular AO/OTA 41-C3 fractures with articular depression of the tibial plateau. In addition, patients who were unable to consent to inclusion and/or would not be able to comply with the study follow-up and treatment regimen where excluded.

### 2.2. Surgical Technique

#### 2.2.1. Patient Positioning

Patients were positioned supine, with their knee semi-extended at approximately 30–45°, supported by a rolled gauze roll under the knee to neutralize the muscle forces, thus reducing any apex-anterior deformities. The biomechanical axis of the lower limb was restored and verified intra-operatively in coronal plane, applying Bowie’s technique with the use of a cautery cable. Additionally, the absence of malrotation in the axial plane and apex-anterior or apex-posterior deformities in the sagittal plane were confirmed before definite fracture fixation; Clemens’ technique, modified by Eckhardt, was applied for intra-operative assessment of rotational alignment [40].

#### 2.2.2. Articular Joint Surface Reduction

In patients with an intra-articular proximal tibial fracture component, anatomical joint surface reduction with compressive fixation was first achieved by percutaneous techniques using either one or two 6.5 mm cannulated screws, or three 3.5 mm subchondral rafting screws, thus converting the fracture from AO/OTA 41-C2.2 to 41-A3. The articular capsule of the knee joint was kept intact, thus avoiding the risk of septic arthritis, subsequent knee stiffness, or permanent debilitating contracture.

#### 2.2.3. Single-Stage Externalized Locked Plating

**Figure 2:**
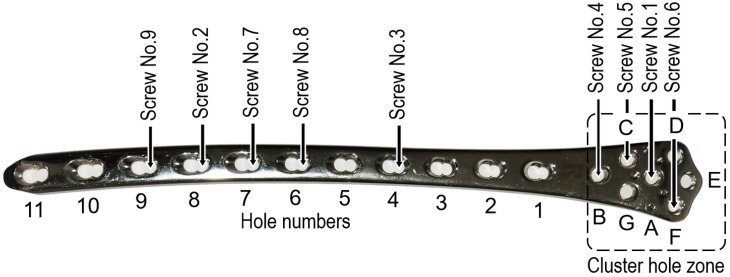
LISS-DF plate with an exemplified sequence of screw insertions into the plate holes.

Eleven-hole LISS-DF plates were used as an external monolateral bridging splint, with a cluster hole zone (holes A to G, Figure 2) offering enough options for adequate locking screw fixation into a short metaphyseal tibial segment. Ipsilateral reversed plates were applied for fixation of proximal, meta-diaphyseal fractures, whereas contralateral, non-reversed plates were used for treatment of distal, meta-diaphyseal fractures. Nine locking screws (No. 1 to No. 9) of appropriate lengths were used, with a specific sequence of their insertion, as visualized in Figure 2. A minimal distance of 15 mm to the articular surface was ensured to avoid penetration into the joint capsule. A provisional subchondral guiding Kirschner (K-) wire was inserted from medial to lateral under C-arm control for this purpose. With a K-wire in the E hole, the plate was then positioned parallel to the anteromedial aspect of the tibia. Additional small stab incisions were cut to assist reduction only if necessary. Manipulating the fragments through the primary wound in open fractures was feasible after debridement and copious irrigation to alleviate the risk of subsequent infection. Manual traction (indirect ligamentotaxis) was applied along the limb axis.

**Figure 3:**
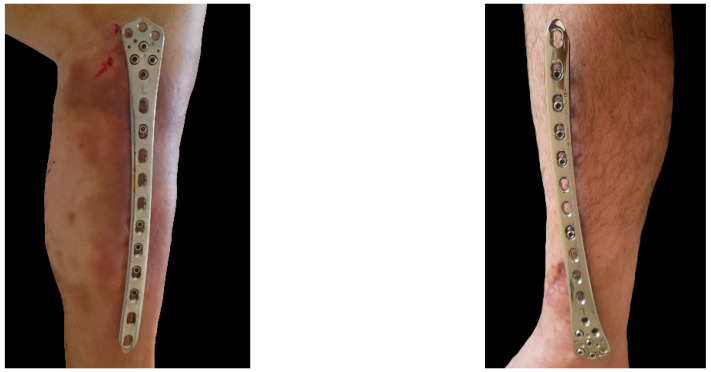
Postoperative photos of patients with a proximal (**left**) and a distal (**right**) meta-diaphyseal fracture fixed via externalized locked plating using either an ipsilateral reversed or a contralateral non-reversed LISS-DF plate, respectively.

Initially, screw No. 1 was inserted in one of the cluster zone holes (A to G, Figure 2), placed parallel to the articular surface and tightened but not yet locked. The hole was selected in a way avoiding penetration of the screw channel into the fracture lines. The screw length was calculated by adding the predefined plate elevation (ranging from 15 to 30 mm) to the measured screw depth, considering thinner or thicker skin coverage and presence or absence of signs for soft tissue edema. Once an optimal alignment was achieved in both frontal and transversal planes, a drill was temporarily inserted into plate hole seven for finetuning of the plate elevation. Screw No. 2 of a corresponding length was then inserted in plate hole eight and tightened but not locked. Thus, a splinting construct with predefined plate elevation, adequate fractured bone alignment, and determined initial bridging span was built. Next, screw No. 3 (drop screw) was inserted and tightened but not locked, next to the diaphyseal end of the fracture zone to minimize the shearing forces at the fracture site and to determine the effective working length, which corresponded to the fracture boundaries. At this stage, a mandatory fluoroscopic control was performed to verify the alignment in frontal and sagittal planes. Following, the inserted three screws were locked and the remaining screws (No. 4 through No. 9) were inserted and locked, too (Figure 3).

#### 2.2.4. Virtual Biomechanical Analysis of Externalized Locked Plating

**Figure 4:**
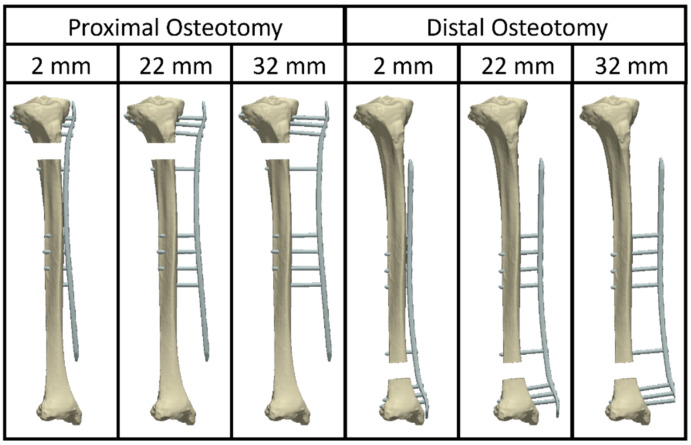
Anterior view of bone-plate constructs with an unstable, meta-diaphyseal, either proximal (**left** images) or distal (**right** images) tibial fracture, mimicked via osteotomizing a 20 mm gap and fixed with three different plate elevations. While the 2 mm plate elevation represents internal fixation, the 22 mm and 32 mm elevations represent externalized locked plating with soft tissue thicknesses of 20 mm and 30 mm, respectively.

To investigate the biomechanical stability of externalized locked plating of proximal and distal unstable tibial fractures, bone-implant constructs with plate elevations representing different soft tissue envelope thicknesses were evaluated and compared using finite element (FE) simulations adopted from previous work (Figure 4) [38].

**Figure 5:**
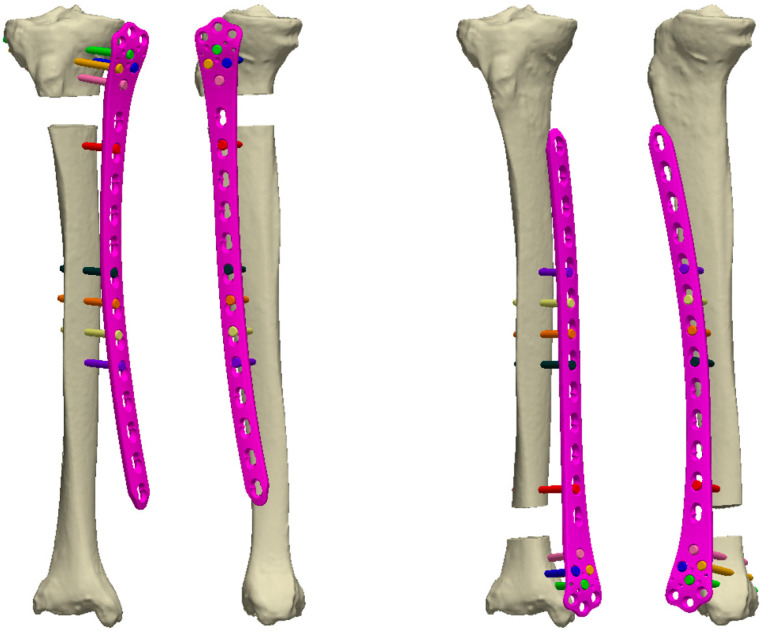
Anterior and medial views of bone-plate constructs with an unstable, meta-diaphyseal, either proximal (**left** images) or distal (**right** images) tibial fracture, mimicked via osteotomizing a 20 mm gap and fixed with a plate elevation of 22 mm representing externalized locked plating with 20 mm soft tissue thickness.

For this purpose, a cadaveric right tibia was scanned via computed tomography (CT, Revolution EVO, GE Healthcare, Chicago, IL, USA). The Hounsfield values were converted to bone mineral density (BMD) using a density phantom (QRM-BDC/6, QRM GmbH, Möhrendorf, Germany). The bone was segmented in the CT image and virtually osteotomized perpendicularly to its long axis to simulate a gap of 20 mm at either the proximal or the distal metaphysis. A computer-aided design (CAD) model of a LISS-DF plate was positioned anteromedially (Figure 5). Three different plate elevations were used to investigate the effect on fracture gap strain at the far cortex. A plate elevation of 2 mm, mimicking internal fixation, was set as control. The other two plate elevations representing different soft tissue envelope thicknesses were defined by lifting the plate by a further 20 mm and 30 mm. CAD models of 5.0 mm locking screws were inserted, with four screws anchoring in the epi-metaphyseal region and five screws anchoring in the midshaft region, with the screw furthest from the fracture being a monocortical screw. Screw lengths were selected based on commercially available lengths.

FE models were meshed with quadratic hexahedral elements using Simpleware software (Simpleware Ltd., Exeter, UK), and the elastic properties of bone elements were mapped based on the underlying BMD values using an established relationship [41]. Stainless steel elastic properties were assigned to the screws and plate (Young’s modulus: 210 GPa; Poission’s ratio: 0.3). Screw-bone and screw-plate interfaces were defined to be bonded. The boundary conditions replicated axial loading with 250 N or 800 N, representing partial or full weight bearing, respectively, as previously described [38]. The FE analyses were performed using Abaqus (V.2019, Simulia, Dassault Systemes, Velizy-Villacoublay, France). Fracture gap movement was measured by tracking the displacement of two nodes on the fracture plane at the far cortex. Fracture gap strain was calculated by dividing the fracture gap movement by the gap width of 20 mm.

### 2.3. Postoperative Protocol for Active Rehabilitation and Plate Removal

From day one to day seven after surgery, patients started with touch weight bearing—walking on the tiptoes of the affected leg with used assistive devices (‘eggshell walking’). In the later phases of callus maturation with clinical evidence of fracture stability, walking with PWB of the limb was performed until radiographic verification of callus formation. After successful radiographic verification of the callus formation by the twelfth postoperative week, full weight bearing (FWB) of the affected limb was started. In the absence of patient complaints, four weeks after FWB without support of crutches and after a new set of orthogonal X-rays, the plate was removed without anesthesia in outpatient clinics.

### 2.4. Data Acquisition and Analysis

All patients in the current study were followed up with orthogonal radiographs at 24 h post-operatively, and at the third, sixth, and twelfth weeks, and at two-month intervals after this. The patients were analyzed in different subgroups based on the categories: age (≤50 and >50 years), fracture location (proximal or distal tibial meta-diaphysis), fracture severity based on the presence or absence of joint involvement (simple or complex), and degree of soft tissue injury (based on the AO soft tissue grading system: mild or severe). Four categories were implemented for assessment of the clinical outcomes: operative time, fracture healing time, functional assessment scores using Hospital for Special Surgery (HSS) (for scoring of the knee joint) and American Orthopedic Foot and Ankle Score (AOFAS) (for scoring of the ankle joint), and knee and ankle joint range of motion (ROM) at the fourth week after surgery and at the final follow-up one month after plate removal. At each consult, the leg was checked for signs of superficial infection and/or screw loosening.

### 2.5. Statistical Analysis

Statistical analysis was performed with the SPSS software package (V.27, IBM, Armonk, NY, USA). The normality of the data distribution was screened and proved with the Shapiro–Wilk test. The Mann–Whitney and Wilcoxon Signed-Rank tests were conducted to detect significant differences among the different subgroups of patients according to the defined categories; the level of significance was set to 0.05 for all statistical tests.

## 3. Results

### 3.1. Clinical Outcomes

The cohort of this prospective study included 18 SSELP applications in 18 patients (15 males and 3 females) aged 53.8 ± 15.9 years (mean ± standard deviation, range 22–85 years). Most of the patients in the cohort (83%) were operated after stabilization of their vital signs and within 24 h of admission. Skeletal traction, for longer than 24 h, was used in 3 (17%) patients who all had multiple injuries, in order to provide provisional fracture stability. All patients were followed up for a period of 21.4 ± 12.3 months (range 14–60 months). The duration of surgery was 33.9 ± 7.1 min (range 20–45 min), with intra-operative X-ray exposure time of 19.6 ± 7.9 s (range 11–43 s). In 17 cases (94%), the fractures healed without complications; the average healing time was 21.1 ± 4.6 weeks (range 12–29 weeks). The clinical outcomes in the different subgroups of patients according to the defined categories are summarized in Table 2.

In patients with simple, extra-articular, multi-fragmentary fractures, the healing time was significantly shorter compared to patients with complex intra-articular fractures (*p* = 0.04). No further significant differences in the clinical outcomes were detected among the subgroups of patients.

**Figure 6:**
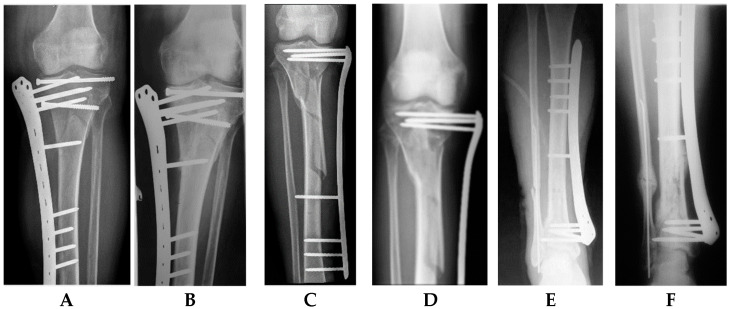
Post-operative (**A**,**C**,**E**) and final follow-up (**B**,**D**,**F**) radiographs of patients with an AO/OTA 41-C2.2 proximal tibial fracture with simple intra-articular involvement of both tibial condyles without intra-articular comminution and impaction (radiographs **A**,**B**), an AO/OTA 41-A3.1 multi-fragmentary proximal tibial extra-articular fracture (radiographs **C**,**D**), and an AO/OTA 43-A2.3 distal tibial multi-fragmentary meta-diaphyseal fracture (radiographs **E**,**F**), all accompanied by significant soft tissue injuries and treated via single-stage externalized locked plating with LISS-DF.

The following complications were observed: 1 delayed union followed by refracturing, 1 case with a knee in 7-degree varus with a leg length discrepancy of 2.5 cm, 1 case with a knee extension contracture due to septic knee arthritis, and 4 cases with loosening of a total of 7 screws. No screw or plate breakage, no deep infection, and no non-unions were observed (Figure 6). Mild, superficial skin irritation around the screw channels of grade 1–2, according to the Checketts–Otterburn classification, with skin granulation of up to 4 mm was identified in 4 patients: 3 patients having hyper granulation of 4–10 mm, and 1 patient with bleeding skin vegetations (hyper granulation with secretion). Plastic repair of the primary wound was necessary in one patient. Fasciotomy was performed in another patient with severe soft tissue injury and an open fracture, although there were no clinical signs of compartment syndrome. A considerable decrease of the post-traumatic soft tissue edema of the injured leg was observed on day one post operation, with a complete disappearance by the end of the first post-operative week, probably due to the active leg mobilization started early.

### 3.2. Functional Outcomes

Among all patients, HSS, AOFAS, and knee flexion increased significantly between the fourth week after surgery and the final follow-up: 89.6 ± 6.6 (range 72–100) to 97.6 ± 3.3 (range 88–100) (*p* < 0.01), 92.3 ± 4.2 (range 84–100) to 98.7 ± 2.0 (range 94–100) (*p* < 0.01), and 105.0 ± 26.5° (range 35–140°) to 130.5 ± 21.6° (range 50–145) (*p* < 0.01), respectively. In contrast, no significant difference was detected between knee joint ROM in extension at the fourth week after surgery (1.7 ± 2.9°, range 0–10°) and the final follow-up (1.3 ± 2.3°, range 0–7°) (*p* = 0.28). Ankle joint ROM in plantar flexion increased significantly between the fourth week after surgery and the final follow-up, from 21.6 ± 5.5° (range 15–30°) to 37.4 ± 9.0° (range 20–50) (*p* < 0.01). Similarly, ankle joint ROM in dorsal flexion increased significantly between the fourth week and the final follow-up, from 12.4 ± 4.5° (range 0–20°) to 18.7± 2.8° (range 10–20) (*p* < 0.01).

The functional outcomes in the different subgroups of patients—according to the defined categories—are summarized in Table 3. In each separate subgroup, all functional outcomes, except knee joint ROM in extension, demonstrated a significant increase between the fourth week after surgery and final follow-up (*p* ≤ 0.04). HSS was significantly lower for patients with complex versus simple fractures at the fourth week after surgery (*p* = 0.04). Knee joint ROM in flexion was significantly higher at the fourth week after surgery for patients suffering distal versus proximal fractures, and at the final follow-up for patients younger versus older than 50 years (*p* < 0.01). Ankle joint ROM in plantar flexion was significantly higher at the fourth week after surgery for patients younger versus older than 50 years (*p* = 0.04). No further significant differences in the functional outcomes were detected among the corresponding subgroups of patients.

**Figure 7:**
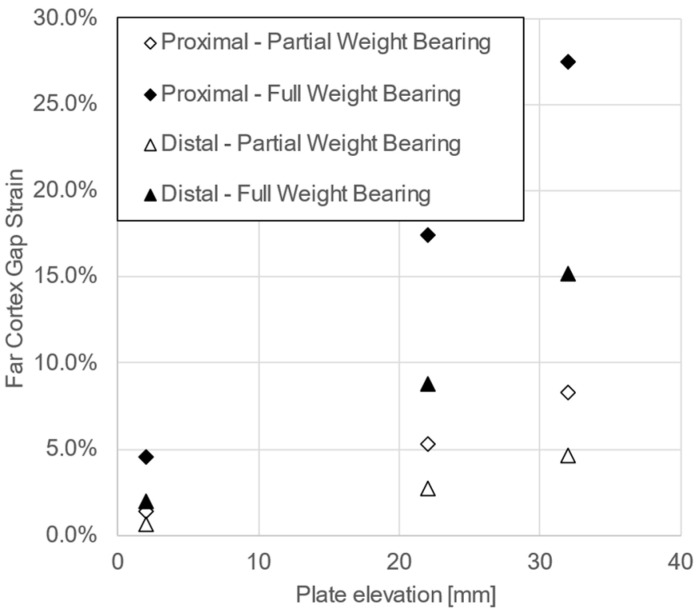
Gap strain at the far cortex as predicted by the FE models of the unstable meta-diaphyseal proximal and distal tibial fractures, mimicked via osteotomizing a 20 mm gap and fixed with three different plate elevations of 2 mm, 22 mm, and 32 mm for partial and full weight bearing scenarios of 250 N and 800 N, respectively.

The virtual biomechanical analysis demonstrated that the interfragmentary movements under PWB in terms of gap strains at the far cortex remained within the range indicated for uneventful secondary fracture healing (Figure 7) [42,43].

## 4. Discussion

This prospective, cohort study focused on the clinical and functional outcomes related to single-stage, externalized locked plating of unstable, proximal intra- and extra-articular and distal extra-articular, meta-diaphyseal tibial fractures. The promising clinical results in the current study were supported by the findings of the performed virtual biomechanical analysis, concluding that the interfragmentary movements during the initial postoperative period remained within the range indicated for uneventful secondary fracture healing.

Adapted for treatment of fractures with specific indications, the technique of externalized, low-profile, locked plating provides a viable and attractive alternative to the conventional external fixation methods and can have higher patient acceptance, while not compromising the stability of fixation [18,25,37]. The technique is characterized by a relative simplicity of performance, smaller soft tissue footprint with less additional soft tissue injury, improved cosmetic appearance, and a more convenient implant removal, thus meeting the criteria for biologic fixation and, therefore, reducing the risk of complications [20,23,24,25,43]. Its use, mostly in one-stage surgery mode, can shorten the overall surgical duration, reduce the intra-operative X-ray exposure time, and decrease both the duration of hospitalization and medical expenses.

The externalized locking plate acts as a buttressing monolateral, external splint. Thus, it ensures relative stability for secondary fracture healing, with easy maintenance of the skin hygiene around the screws, being less prominent and aesthetically acceptable for the patient [6]. Due to the low profile, the plate can be placed very close to the skin and then easily concealed under patient’s clothing, thus overcoming the disadvantages of conventional external fixators and eliminating their drawback of causing a greater deal of inconvenience and discomfort [32,44].

Commonly, both techniques, using either conventional external fixators or externalized locking plates, rely on fixed-angle stabilization—between the pins and the side bar of the fixator or between the screws and the plate—to span the fracture at a fixed distance over the bone surface without considerable additional irritation of the periosteal blood supply [37,45]. Moreover, to achieve a suitable mechanical environment for secondary bone healing, both these techniques allow a minimally invasive osteosynthesis and modulation of construct stiffness to a certain degree [37,46].

However, despite the implied similarity between the techniques, some substantial differences primarily pertain regarding the methods for stiffness control, as well as the extent and range to which the stiffness can be modulated [37]. Whereas conventional external fixators can be applied in a wide variety of frame configurations, allowing control of the stiffness over a broad range via changing both inter-pin distances and distances of the pins to the fracture line, the range over which the stiffness of an externalized locking plate construct can be modulated is rather narrower—compared with external fixators—as there is a limited number of screw holes that may be occupied [37,47]. Furthermore, in contrast to conventional external fixations, increasing the number of rods to enhance the construct stiffness is not possible for the externalized locked plating [37].

Another substantial difference between the two techniques is that the externalized locked plating requires a good reduction prior to fixation due to the limited adjustments that can be made after that.

The higher flexibility of manipulation due to the adjustable clamps, together with the standardized implantation technique, are advantages of the conventional external fixator. Hence, it is worth noting that the externalized locked plating has the following disadvantage: it can be hard to position the plate and adjust the direction of each individual screw because of both the locking mechanism and the highly accurate reduction at the fracture site that should be achieved and maintained [20]. Variable-angle locking technologies offer some more freedom in this regard by allowing individual screw orientation to a certain extent.

Considering this, the surgical technique related to single-stage externalized locked plating was adapted in the current study by: (1) achieving appropriate fracture reduction, (2) provisional optimal positioning and alignment of the plate in both the coronal and sagittal planes, together with the predefined plate elevation and determined initial bridging span, (3) initial sequential insertion and tightening of all screws in the plate holes without locking, (4) fluoroscopic control to verify the alignment in the coronal and sagittal planes, and (5) locking of the inserted screws in a predefined sequence.

While external fixators are mostly used in emergency situations for initial treatment of fractures with severe soft tissue injuries, as well as in multiple trauma patients with required emergent ‘damage control’ fixation, externalized locking plates are meant to apply for definitive fracture treatment. Distal tibial fractures often require application of a spanning external fixator secondary to associated soft tissue swelling. Unfortunately, closed reduction performed at the time of external fixator application does not provide sufficient reconstruction of the articular surface. Delayed fracture healing and soft tissue complications are frequently seen, especially in cases with extensive soft tissue dissection and periosteal debridement during open reduction and joint reconstruction. Similar to fractures of the distal tibia, the treatment of proximal tibial fractures can be challenging as a result of extensive soft tissue damage and the special biomechanical situation at the proximal tibia. In contrast, during externalized locked plating, when both fracture reduction and plate positioning are appropriate, the proximal and distal locking holes are secured with locking screws.

The operative time in the current study—being similar for unstable proximal and distal meta-diaphyseal simple and complex fractures—was comparable with previous reports [23,24]. The fracture healing time in the present work—being significantly different for patients with intra- and extra-articular fractures—was within the reported union ranges in previous studies [6,21,24]. The current HSS and AOFAS scores, assessed at the fourth week after surgery and the final follow-up, were in agreement with previous work [21]. For all patients in the current cohort, the plate elevation was equal to or less than 30 mm to keep a stable fixation according to previous studies [33,48].

In a prospective series of 25 patients with segmental tibial fractures, treated via a 2-stage procedure involving supercutaneous plating, the reported rates of excellent and good functional outcomes were 84% and 16%, respectively [22]. The current study demonstrated good and excellent functional outcomes among all patients. Thus, it is in agreement with another prospective report on 12 patients suffering tibial fractures with compromised soft tissue envelopes, treated with definitive externalized locked plating, which concluded that all patients had excellent or good functional outcomes [20]. In a clinical study review on a total of 254 patients treated with externalized locked plating, only 2 cases of non-union with a mean rate of bone union 99.2% (range 95.7–100%) were reported along with a low implant complication rate—only 1 broken plate, 5 cases with 6 loose screws, and 3 cases with 4 broken screws [6]. The bone union rate in the current study was 100%, with no screw and plate breakage and 4 cases with 7 loose screws. The results from the virtual biomechanical analysis confirmed previous conclusions that partial weight bearing was possible for fractured tibiae fixed by supercutaneous plating with plate elevation of up to 30 mm when the patients’ activities were considered with caution during the early postoperative phase [33,35,38].

The limitations of the present study include the generalizability of the findings, given the small cohort size of patients treated in a single medical center by a single surgeon, the risk of bias, and the absence of a control group. Despite this, it was possible to detect significant differences among the subgroups of patients. Indeed, an important advantage of the study is the inclusion of patients with both proximal (intra- and extra-articular) and distal (extra-articular) meta-diaphyseal tibial fractures. Another important advantage is the high acceptance of the externalized locked plating by the patients. Moreover, the fact that all procedures and follow-up assessments were conducted by a single surgeon at a single center with a consistent technique adds to the validity of the results. Only one implant was used, and results may vary when using other locking plates.

## 5. Conclusions

Single-stage externalized locked plating of specifically targeted tibial fractures provides adequate fixation, generating promising clinical results, and represents an attractive alternative to the conventional methods of external fixation when the inclusion criteria and rehabilitation protocol are strictly followed. Further experimental studies and randomized multicentric clinical trials with larger series of patients are necessary to pave the way of its use in clinical practice.

## Figures and Tables

**Table 1:** Patients and fracture characteristics.

Classification	Type/Grade	Number of Patients	Gender
AO/OTA fracture classification	41-C2.2	7	M
	41-C2.3	1	F
	41-A3.1	2	M
	41-A3.1	2	F
	42-C3j	2	M
	43-A2.1	1	M
	43-A2.3	3	M
AO soft tissue grading system	IO2	7	M
	IO2	3	F
	IO3	6	M
	IO4	2	M

**Table 2:** Clinical outcomes in the subgroups of patients according to the categories age, fracture location, fracture complexity, and soft tissue injury, presented in terms of mean value, standard deviation and range, together with the *p*-values from the corresponding subgroups comparisons.

Categories	Subgroups	Operative Time (Minutes)	Healing Time (Weeks)
Age	≤50 years	31.4 ± 7.1 (25–45)	20.7 ± 6.1 (12–28)
	>50 years	34.4 ± 6.6 (20–45)	20.9 ± 3.6 (16–29)
	*p*-value	0.26	0.93
Fracture location	Proximal	32.4 ± 6.8 (20–45)	21.8 ± 3.6 (16–28)
	Distal	34.8 ± 6.9 (25–45)	19.0 ± 6.1 (12–29)
	*p*-value	0.38	0.25
Fracture severity	Simple	34.3 ± 5.5 (25–42)	18.4 ± 2.9 (14–22)
	Complex	32.6 ± 7.6 (20–45)	22.4 ± 4.9 (12–29)
	*p*-value	0.42	0.04
Soft tissue injury	Mild	34.0 ± 7.1 (20–45)	21.2 ± 5.3 (12–29)
	Severe	32.2 ± 6.6 (20–45)	21.1 ± 3.8 (14–26)
	*p*-value	0.46	0.76

**Table 3:** Functional outcomes in the subgroups of patients according to the categories age, fracture location, fracture complexity, and soft tissue injury, presented in terms of mean value and *p*-values from the corresponding subgroups and time comparisons at the fourth week after surgery (4 weeks) and at the final follow-up (Final FU).

Categories	Subgroups	HSS			AOFAS			ROM knee (°)						ROM Ankle Flexion (°)					
								Flexion			Extension			Plantar			Dorsal		
		4 Weeks	Final FU	*p*-Value	4 Weeks	Final FU	*p*-Value	4 Weeks	Final FU	*p*-Value	4 Weeks	Final FU	*p*-Value	4 Weeks	Final FU	*p*-Value	4 Weeks	Final FU	*p*-Value
Age	≤50 years	88.7	98.6	0.01	93.6	99.1	0.03	113.6	142.1	0.03	0.4	0.4	0.99	25.0	40.0	0.02	13.6	18.6	0.02
	>50 years	89.9	96.8	<0.01	91.4	98.3	<0.01	97.7	121.4	<0.01	2.7	2.0	0.18	19.6	35.4	<0.01	11.4	18.5	<0.01
	*p*-value	0.72	0.54	–	0.29	0.28	–	0.15	<0.01	–	0.13	0.38	–	0.04	0.28	–	0.13	0.79	–
Fracture location	Proximal	87.5	97.2	<0.01	92.0	92.8	<0.01	92.1	125.0	<0.01	2.2	1.7	0.19	22.1	35.4	<0.01	12.1	18.3	<0.01
	Distal	93.3	98.2	0.04	98.1	98.7	0.03	127.5	138.3	0.04	0.8	0.8	0.99	20.8	40.8	0.02	12.5	19.2	0.03
	*p*-value	0.18	0.55	–	0.98	0.89	–	<0.01	0.29	–	0.55	0.62	–	0.68	0.29	–	0.82	0.75	–
Fracture complexity	Simple	93.9	98.7	0.04	91.7	98.3	0.02	114.3	132.1	0.04	0.7	0.7	0.98	21.4	37.1	0.02	11.4	18.6	0.02
	Complex	86.6	96.7	<0.01	92.4	98.8	<0.01	97.2	127.7	<0.01	2.4	1.8	0.41	21.8	37.2	<0.01	12.7	18.7	<0.01
	*p*-value	0.04	0.21	–	0.38	0.42	–	0.18	0.79	–	0.42	0.48	–	0.86	0.93	–	0.42	0.79	–
Soft tissue injury	Mild	91.0	98.5	<0.01	93.0	98.6	<0.01	103.5	131.0	<0.01	2.2	1.5	0.32	22.0	37.0	<0.01	12.0	19.0	<0.01
	Severe	85.7	96.2	0.01	91.4	98.5	0.02	104.4	127.5	0.02	1.2	1.2	0.99	21.2	37.5	0.01	12.5	18.1	0.01
	*p*-value	0.52	0.27	–	0.46	0.63	–	0.57	0.32	–	0.46	0.90	–	0.76	0.70	–	0.63	0.83	–

## Data Availability

The datasets analyzed during the performance of the current study are available from the corresponding author on reasonable request. In order to comply with the requirements of the Ethics Committee, the image set is not available for request due to data privacy policies.

## References

[1] Catagni M.A., Ottaviani G., Maggioni M. (2007). Treatment strategies for complex fractures of the tibial plateau with external circular fixation and limited internal fixation. J. Trauma.

[2] Gosling T., Schandelmaier P., Muller M., Hankemeier S., Wagner M., Krettek C. (2005). Single lateral locked screw plating of bicondylar tibial plateau fractures. Clin. Orthop. Relat. Res..

[3] Bove F., Sala F., Capitani P., Thabet A.M., Scita V., Spagnolo R. (2018). Treatment of fractures of the tibial plateau (Schatzker VI) with external fixators versus plate osteosynthesis. Injury.

[4] Yang S.W., Tzeng H.M., Chou Y.J., Teng H.P., Liu H.H., Wong C.Y. (2006). Treatment of distal tibial metaphyseal fractures: Plating versus shortened intramedullary nailing. Injury.

[5] Zelle B.A., Bhandari M., Espiritu M., Koval K.J., Zlowodzki M. (2006). Treatment of distal tibia fractures without articular involvement: A systematic review of 1125 fractures. J. Orthop. Trauma.

[6] Luo P., Xu D., Wu J., Chen Y.H. (2017). Locked plating as an external fixator in treating tibial fractures: A PRISMA-compliant systematic review. Medicine (Baltimore).

[7] Lau T.W., Leung F., Chan C.F., Chow S.P. (2008). Wound complication of minimally invasive plate osteosynthesis in distal tibia fractures. Int. Orthop..

[8] Scolaro J.A., Broghammer F.H., Donegan D.J. (2016). Intramedullary Tibial Nail Fixation of Simple Intraarticular Distal Tibia Fractures. J. Orthop. Trauma.

[9] Kalinterakis G., Koutras A., Syllaios A., Michalakeas N., Lytras D., Tsilikis I. (2019). The evolution and impact of the “damage control orthopedics” paradigm in combat surgery: A review. Eur. J. Orthop. Surg. Traumatol..

[10] Hasenboehler E., Rikli D., Babst R. (2007). Locking compression plate with minimally invasive plate osteosynthesis in diaphyseal and distal tibial fracture: A retrospective study of 32 patients. Injury.

[11] Janssen K.W., Biert J., van Kampen A. (2007). Treatment of distal tibial fractures: Plate versus nail: A retrospective outcome analysis of matched pairs of patients. Int. Orthop..

[12] Li B., Yang Y., Jiang L.S. (2015). Plate fixation versus intramedullary nailing for displaced extra-articular distal tibia fractures: A system review. Eur. J. Orthop. Surg. Traumatol..

[13] Marsh J.L., Bonar S., Nepola J.V., Decoster T.A., Hurwitz S.R. (1995). Use of an articulated external fixator for fractures of the tibial plafond. J. Bone Joint. Surg. Am..

[14] Nork S.E., Schwartz A.K., Agel J., Holt S.K., Schrick J.L., Winquist R.A. (2005). Intramedullary nailing of distal metaphyseal tibial fractures. J. Bone Joint. Surg. Am..

[15] Sitnik A.A., Beletsky A.V. (2013). Minimally invasive percutaneous plate fixation of tibia fractures: Results in 80 patients. Clin. Orthop. Relat. Res..

[16] Fang X., Jiang L., Wang Y., Zhao L. (2012). Treatment of Gustilo grade III tibial fractures with unreamed intramedullary nailing versus external fixator: A meta-analysis. Med. Sci. Monit..

[17] Kerkhoffs G.M., Kuipers M.M., Marti R.K., Van der Werken C. (2003). External fixation with standard AO-plates: Technique, indications, and results in 31 cases. J. Orthop. Trauma.

[18] Kloen P. (2009). Supercutaneous plating: Use of a locking compression plate as an external fixator. J. Orthop. Trauma.

[19] Marti R.K., van der Werken C. (1991). The AO-plate for external fixation in 12 cases. Acta Orthop. Scand..

[20] Qiu X.S., Yuan H., Zheng X., Wang J.F., Xiong J., Chen Y.X. (2014). Locking plate as a definitive external fixator for treating tibial fractures with compromised soft tissue envelop. Arch. Orthop. Trauma Surg..

[21] Ma C.H., Wu C.H., Jiang J.R., Tu Y.K., Lin T.S. (2017). Metaphyseal locking plate as an external fixator for open tibial fracture: Clinical outcomes and biomechanical assessment. Injury.

[22] Ma C.H., Tu Y.K., Yeh J.H., Yang S.C., Wu C.H. (2011). Using external and internal locking plates in a two-stage protocol for treatment of segmental tibial fractures. J. Trauma.

[23] Zhang J., Ebraheim N., Li M., He X., Liu J., Zhu L., Yu Y. (2015). External fixation using femoral less invasive stabilization system plate in tibial proximal metaphyseal fracture. Clin. Orthop. Surg..

[24] Zhang J.W., Ebraheim N.A., Li M., He X.F., Schwind J., Zhu L.M., Yu Y.H. (2016). Distal tibial fracture: An ideal indication for external fixation using locking plate. Chin. J. Traumatol..

[25] Zhou Y., Wang Y., Liu L., Zhou Z., Cao X. (2015). Locking compression plate as an external fixator in the treatment of closed distal tibial fractures. Int. Orthop..

[26] Apivatthakakul T., Sananpanich K. (2007). The locking compression plate as an external fixator for bone transport in the treatment of a large distal tibial defect: A case report. Injury.

[27] Ebraheim N.A., Carroll T., Hanna M., Zhang J., Liu J. (2014). Staged treatment of proximal tibial fracture using external locking compression plate. Orthop. Surg..

[28] Khatod M., Botte M.J., Hoyt D.B., Meyer R.S., Smith J.M., Akeson W.H. (2003). Outcomes in open tibia fractures: Relationship between delay in treatment and infection. J. Trauma.

[29] Hidayat L., Triangga A.F.R., Cein C.R., Irfantian A., Rahayu B.F.P., Resubun A.P., Magetsari R. (2022). Low profile external fixation using locking compression plate as treatment option for management of soft tissue problem in open tibia fracture grade IIIA: A case series. Int. J. Surg. Case Rep..

[30] Ma C.H., Wu C.H., Tu Y.K., Lin T.S. (2013). Metaphyseal locking plate as a definitive external fixator for treating open tibial fractures--clinical outcome and a finite element study. Injury.

[31] Zhang J., Ebraheim N.A., Li M., He X., Liu J., Zhu L., Yu Y., Siddiqui S. (2015). External Fixation Using a Locking Plate: A Reliable Way in Treating Distal Tibial Fractures. J. Orthop. Trauma.

[32] Tulner S.A., Strackee S.D., Kloen P. (2012). Metaphyseal locking compression plate as an external fixator for the distal tibia. Int. Orthop..

[33] Kanchanomai C., Phiphobmongkol V. (2012). Biomechanical evaluation of fractured tibia externally fixed with an LCP. J. Appl. Biomech..

[34] Liu W., Yang L., Kong X., An L., Hong G., Guo Z., Zang L. (2017). Stiffness of the locking compression plate as an external fixator for treating distal tibial fractures: A biomechanics study. BMC Musculoskelet. Disord..

[35] Zhang J., Ebraheim N., Li M., He X., Schwind J., Liu J., Zhu L. (2015). External fixation using locking plate in distal tibial fracture: A finite element analysis. Eur. J. Orthop. Surg. Traumatol..

[36] Blažević D., Kodvanj J., Adamović P., Vidović D., Trobonjača Z., Sabalić S. (2022). Comparison between external locking plate fixation and conventional external fixation for extraarticular proximal tibial fractures: A finite element analysis. J. Orthop. Surg. Res..

[37] Ang B.F.H., Chen J.Y., Yew A.K.S., Chua S.K., Chou S.M., Chia S.L., Koh J.S.B., Howe T.S. (2017). Externalised locking compression plate as an alternative to the unilateral external fixator: A biomechanical comparative study of axial and torsional stiffness. Bone Joint. Res..

[38] Makelov B., Silva J.D., Apivatthakakul T., Gueorguiev B., Varga P. (2019). Externalized locked plating of unstable high energy proximal tibial fractures. A FEA study. Bul. J. Orthop. Trauma.

[39] Makelov B., Gueorguiev B., Apivatthakakul T. (2019). Definitive external plate stabilization with metaphyseal locking plate LISS-DF in multiple trauma patient with ‘‘floating knee’’ injury. Bul. J. Orthop. Trauma..

[40] Eckardt H., Morgenstern M., Cadosch D., Stoffel K. (2021). Flouroscopic Control of Tibial Torsion After Intramedullary Nailing: A Technical Trick. J. Orthop. Trauma.

[41] Dragomir-Daescu D., Op Den Buijs J., McEligot S., Dai Y., Entwistle R.C., Salas C., Melton L.J., Bennet K.E., Khosla S., Amin S. (2011). Robust QCT/FEA models of proximal femur stiffness and fracture load during a sideways fall on the hip. Ann. Biomed. Eng..

[42] Perren S.M. (1979). Physical and biological aspects of fracture healing with special reference to internal fixation. Clin. Orthop. Relat. Res..

[43] Perren S.M. (2002). Evolution of the internal fixation of long bone fractures. The scientific basis of biological internal fixation: Choosing a new balance between stability and biology. J. Bone Joint. Surg. Br..

[44] Possley D.R., Burns T.C., Stinner D.J., Murray C.K., Wenke J.C., Hsu J.R. (2010). Temporary external fixation is safe in a combat environment. J. Trauma.

[45] Moss D.P., Tejwani N.C. (2007). Biomechanics of external fixation: A review of the literature. Bull. NYU Hosp. Jt. Dis..

[46] Schmal H., Strohm P.C., Jaeger M., Südkamp N.P. (2011). Flexible fixation and fracture healing: Do locked plating ‘internal fixators’ resemble external fixators?. J. Orthop. Trauma.

[47] MacLeod A.R., Simpson A.H., Pankaj P. (2016). Age-related optimization of screw placement for reduced loosening risk in locked plating. J. Orthop. Res..

[48] Ahmad M., Nanda R., Bajwa A.S., Candal-Couto J., Green S., Hui A.C. (2007). Biomechanical testing of the locking compression plate: When does the distance between bone and implant significantly reduce construct stability?. Injury.

